# Signals of Systemic Immunity in Plants: Progress and Open Questions

**DOI:** 10.3390/ijms19041146

**Published:** 2018-04-10

**Authors:** Attila L. Ádám, Zoltán Á. Nagy, György Kátay, Emese Mergenthaler, Orsolya Viczián

**Affiliations:** 1 Plant Protection Institute, Centre for Agricultural Research, Hungarian Academy of Sciences, 15 Herman Ottó út, H-1022 Budapest, Hungary; katay.gyorgy@agrar.mta.hu (G.K.); mergenthaler.emese@agrar.mta.hu (E.M.); viczian.orsolya@agrar.mta.hu (O.V.); 2 Phytophthora Research Centre, Department of Forest Protection and Wildlife Management, Faculty of Forestry and Wood Technology, Mendel University in Brno, Zemědělská 3, 613 00 Brno, Czech Republic; zoltan.nagy@mendelu.cz

**Keywords:** *Arabidopsis*, azelaic acid, glycerol-3-phosphate, light dependent signalling, methyl salicylate, *N*-hydroxypipecolic acid, pipecolic acid, salicylic acid, SAR signalling, spectral distribution of light, tobacco

## Abstract

Systemic acquired resistance (SAR) is a defence mechanism that induces protection against a wide range of pathogens in distant, pathogen-free parts of plants after a primary inoculation. Multiple mobile compounds were identified as putative SAR signals or important factors for influencing movement of SAR signalling elements in *Arabidopsis* and tobacco. These include compounds with very different chemical structures like lipid transfer protein DIR1 (DEFECTIVE IN INDUCED RESISTANCE1), methyl salicylate (MeSA), dehydroabietinal (DA), azelaic acid (AzA), glycerol-3-phosphate dependent factor (G3P) and the lysine catabolite pipecolic acid (Pip). Genetic studies with different SAR-deficient mutants and silenced lines support the idea that some of these compounds (MeSA, DIR1 and G3P) are activated only when SAR is induced in darkness. In addition, although AzA doubled in phloem exudate of *tobacco mosaic virus* (TMV) infected tobacco leaves, external AzA treatment could not induce resistance neither to viral nor bacterial pathogens, independent of light conditions. Besides light intensity and timing of light exposition after primary inoculation, spectral distribution of light could also influence the SAR induction capacity. Recent data indicated that TMV and CMV (*cucumber mosaic virus*) infection in tobacco, like bacteria in *Arabidopsis,* caused massive accumulation of Pip. Treatment of tobacco leaves with Pip in the light, caused a drastic and significant local and systemic decrease in lesion size of TMV infection. Moreover, two very recent papers, added in proof, demonstrated the role of FMO1 (FLAVIN-DEPENDENT-MONOOXYGENASE1) in conversion of Pip to *N*-hydroxypipecolic acid (NHP). NHP systemically accumulates after microbial attack and acts as a potent inducer of plant immunity to bacterial and oomycete pathogens in *Arabidopsis*. These results argue for the pivotal role of Pip and NHP as an important signal compound of SAR response in different plants against different pathogens.

## 1. Introduction

Systemic acquired resistance (SAR) is an inducible defence mechanism that provides protection in distant, pathogen-free parts of plants against a broad range of pathogens. In practice, SAR has been recognised as a strategy to control plant pathogens because of its stability [1], long-lasting effectiveness [2], and transgenerational effect [3]. In the latter case, defence mechanisms are induced faster in the progeny after pathogen infection. The plant “immune” memory involved in the inheritance of SAR has probably an epigenetic character that effects the patterns of DNA methylation at the promoters of defence-related genes [3]. However, it is important to note that the mechanism via which DNA methylation regulates SAR within a single generation may differ from transgenerational SAR responses [4]. Tissues exhibiting SAR in the distant, pathogen-free parts of plants, display a “prepared” state (defence priming) associated with faster and stronger defence mechanisms [5,6,7]. This memory is based on posttranslational modification of histone and results in changes of the structure of chromatin [8]. This regulation is an integrated part of biotic and abiotic stress responses and is called the “histone code” [9].

**Figure 1:**
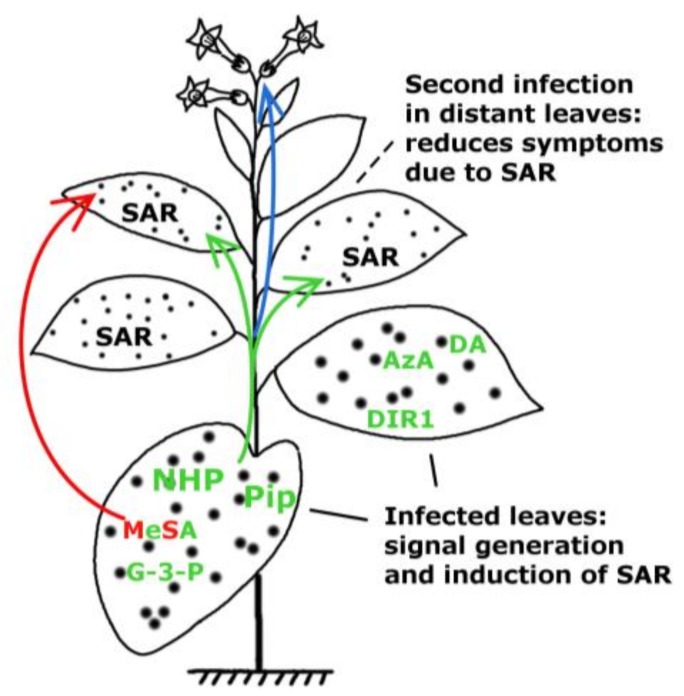
Development of systemic acquired resistance (SAR). Putative SAR signal molecules: methyl salicylate (MeSA), lipid transfer protein DIR1 (DEFECTIVE IN INDUCED RESISTANCE1), dehydroabietinal (DA), glycerol-3-phosphate (G3P) or G3P-dependent factor, azelaic acid (AzA), pipecolic acid (Pip) and *N*-hydroxypipecolic acid (NHP) move from the infected organ (leaves) to pathogen-free distant parts of the plant where they induce SAR against biotrophic and hemibiotrophic pathogens (note the limited symptom expression in the distant, systemic leaves indicated by small spots). Pip and NHP are highlighted. Green and red arrows indicate the movement of signal molecules via phloem transport or the air (putative volatile compounds for inplant or interplant airborne signals), respectively. Blue arrow indicates transgenerational SAR where the epigenetic information is inherited and present in the next generation (re-drawn and modified from [28]). For chemical structures of signal compounds see Figure 2.

**Figure 2:**
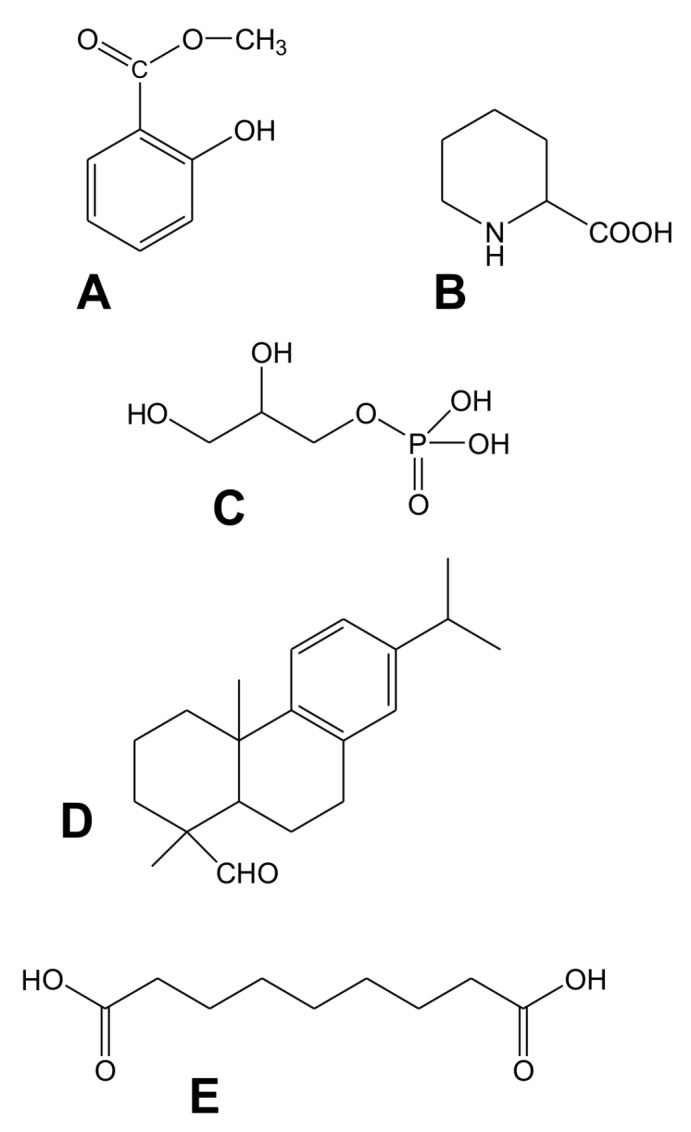
Chemical structures of signal compounds participating in SAR induction. (**A**) Phenolic compound, methyl-salicylate; (**B**) Non-protein amino acid, pipecolic acid; (**C**) Glycerol-3-phosphate; (**D**) Geranylgeranyl diphosphate derived dehydroabietinal; (**E**) Dicarboxylic acid, azelaic acid.

The SAR response can be divided into four steps in which signal generation in primary infected leaves and signal movement (Figure 1) into distant organs (leaves) are both important steps [10]. To date, several mobile compounds have been identified as putative SAR signals or important factors for movement of long-distance SAR signals in *Arabidopsis* and tobacco. These include methyl salicylate (MeSA) [11,12,13] the lipid transfer protein DIR1 (DEFECTIVE IN INDUCED RESISTANCE1) [14,15], dehydroabietinal (DA) [16], azelaic acid (AzA) [17,18], glycerol-3-phosphate dependent factor (G3P) [19,20] and the lysine catabolite amino acid, pipecolic acid (Pip) [21,22]. Chemical structures of signal compounds of SAR are shown in Figure 2. As a last step, after signal perception, the manifestation of SAR (defence priming) in distant leaves (Figure 1) is associated with a massive transcriptional and metabolic reprogramming [7,20,23]. In *Arabidopsis,* this requires several factors in distant, pathogen free leaves: pipecolic acid (Pip) and salicylic acid (SA) accumulation, expression of *ALD1* (*AGD2-LIKE DEFENSE RESPONSE PROTEIN1*), *FMO1* (*FLAVIN-DEPENDENT*-*MONOOXYGENASE1*), *PAD4* (*PHYTOALEXIN DEFICIENT4*), *ICS1* (*ISOCHORISMATE SYNTHASE1*), *FLD* (*FLOWERING LOCUS D1*), *EDS1* (*ENHANCED DISEASE SUSCEPTIBILITY1*), and *SnRK2.8* (*SNF1-RELATED PROTEIN KINASE2.8*) genes, most of them are components of the SA-amplification loop. SA-dependent activation of a transcriptional regulator, *NPR1* (*NON-EXPRESSOR OF PR GENES1*) is also necessary for defence priming [16,21,23,24,25,26,27]. However, it is important to note that defence priming and signal amplification are dependent on systemic Pip accumulation and Pip orchestrates SA-dependent and SA-independent priming of plant responses in a *FMO1*-dependent manner [21,23].

Former studies also suggested that the SAR-inducing activity in phloem sap of *Arabidopsis* and cucumber induced by different pathogens was effective in other plants [16,20,29], thus indicating that the mobile SAR signal(s) is neither plant- nor pathogen-specific. However, considering recent data about multiple signal mechanisms with diverse chemical substances described mainly in *Arabidopsis*-bacteria plant-pathogen systems it is reasonable to test whether this multiple signalling is due to environmental or some other circumstantial factors. It is especially true for tobacco where less experimental data is available and a virus-inducible SAR response could be also studied by using a computer-assisted method for symptoms evaluation and statistical analysis [28,30,31,32].

Plants possess a sophisticated and structured immune response against invading pathogens. The first layer in plant defence is pattern triggered immunity (PTI) which is induced by specific microbe-associated molecular patterns (MAMPs) which are recognized by pattern recognition receptors (PRRs) at the plasma membrane. In addition, endogenous molecules, like extracellular ATP, so-called danger-associated molecular pattern (DAMP) can also induce plant defence response [33,34]. The specific role of PTI in local antiviral defence has been recently seen in *Arabidopsis*. PRR coreceptor kinase mutant, *bak1/serk3* (brassinosteroid insensitive1 (bri1)-associated receptor kinase1 or somatic emryogenesis receptor-like kinase3, *serk3*) and other *SERK* gene family mutants [35] have increased susceptibility to different RNA viruses (note that mutant genotypes are indicated by small letters in italics of the corresponding wild type gene all over the paper). SERKs are a family of closely related leucine-rich repeat receptor kinases involved in different plant signalling routes where they act in a partially redundant manner as coreceptors for the respective PRRs [36]. *BAK1* is also required for PTI responses to bacterial MAMPs such as flagellin-derived flg-22 and translation elongation factor-derived peptide, elf18. In addition, *BAK1* and *SERK4* play a role in peptide elicitor signalling and cell death control [37,38]. Connecting PTI signalling with SAR, Mishina and Zeier [39] found that SAR could be induced by bacterial MAMPs (flagellin and LPS) against bacteria in *Arabidopsis* and the extent of tissue necrosis at the site of inoculation by virulent or avirulent bacteria is dispensable for the SAR response. However, to date, the chemical nature of elicitors of viral origin or virus inducible elicitors of PTI are unknown.

**Figure 3:**
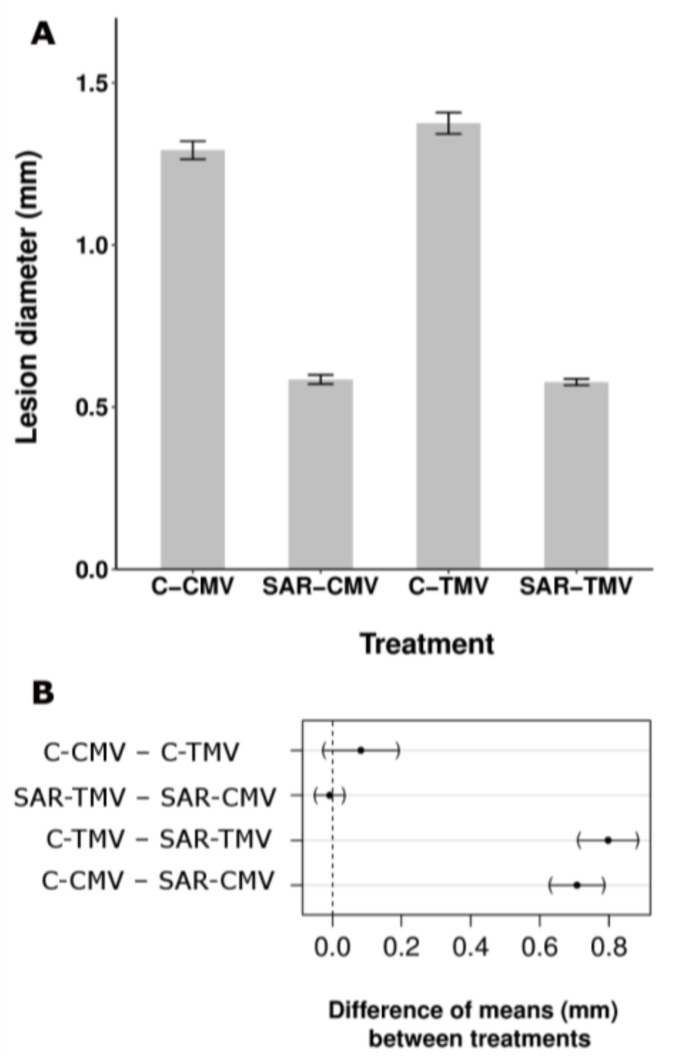

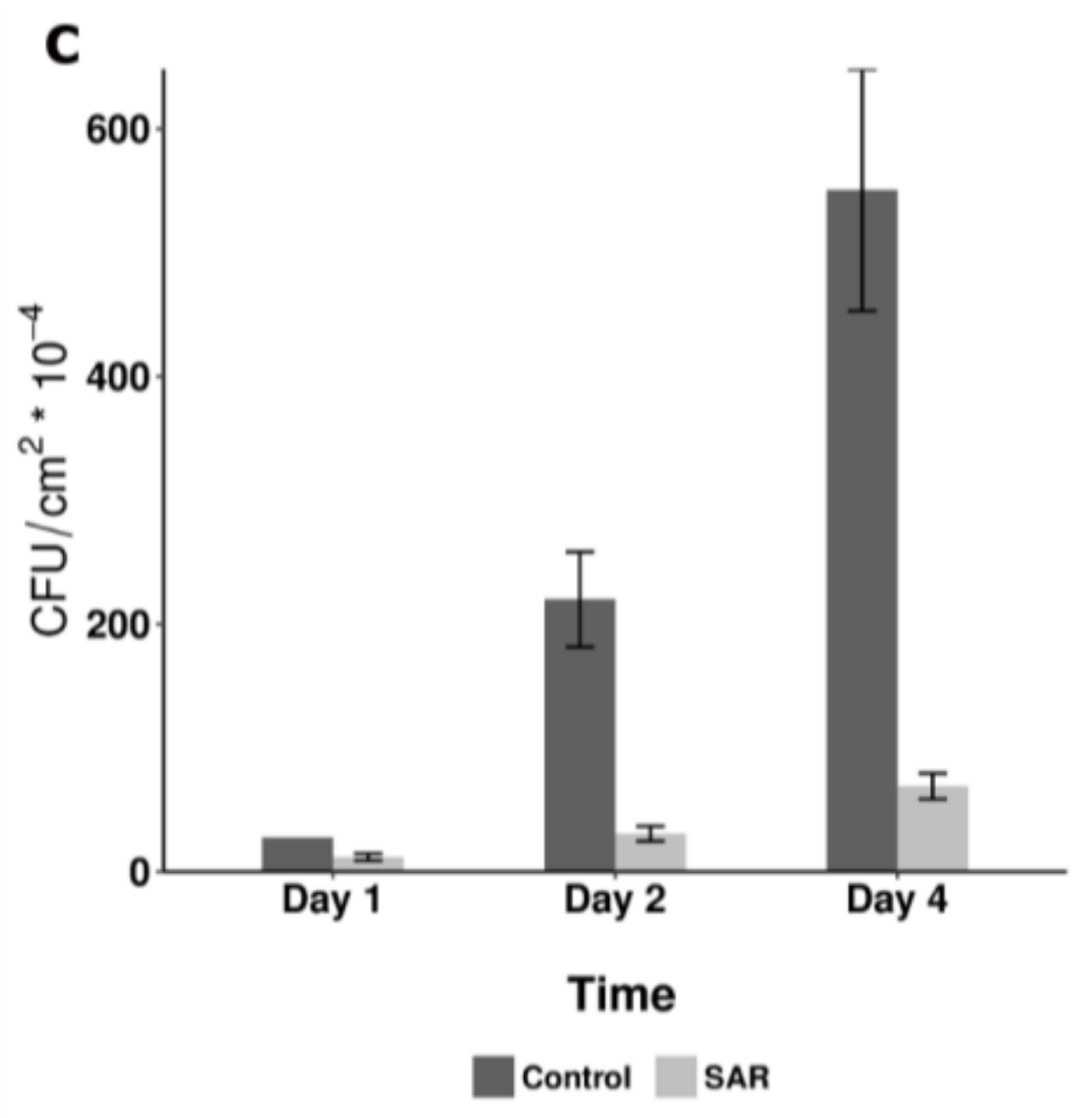
Aspecific feature of SAR induction in tobacco (*N. tabacum* cv. Xanthi nc). TMV induced SAR response was effective not only against TMV and another viral (cucumber mosaic virus, CMV necrotic strain) (**A**,**B**) but bacterial (*Pseudomonas syringae* pv. *tabaci*) (**C**) pathogens as well. SAR was induced by TMV infection as described earlier [31] and 10 days later the 5th and 6th leaves were challenged by CMV (SAR-CMV) and TMV (SAR-TMV) as compared to controls (C-CMV and C-TMV) (half leaves of each case) (**A**) or by bacterial suspension (10^5^ CFU mL^−1^) (**C**) in triplicates per treatments. (**B**) Multiple comparison of means of selected treatments in (**A**). Dots represent the difference of the estimated means between treatments, brackets flank the 95% confidence intervals. The difference is considered significant if the confidence interval does not contain the 0, represented by a vertical dashed line [31]. (**C**) The SAR induction was also checked by TMV challenge inoculation (showing 69.6% significant inhibition of TMV lesion size). The TMV induced SAR caused time-dependent and significant (generalized linear model) decrease of bacterial multiplication

Therefore, we checked the specificity of TMV-induced SAR response in tobacco of which we have no or limited information (Figure 3) [40]. TMV infection resulted in SAR response not only against TMV [2] (Figure 3A) but also against bacterial (*P. syringae* pv. *tabaci*) (Figure 3C) and other viral (necrotic strain of cucumber mosaic virus, CMV Ns) [41] (Figure 3A) pathogens. SAR response induced by TMV was as much as effective against TMV as CMV (58.0% and 54.7% inhibition of mean lesion size as compared to control plants, respectively) (Figure 3A,B). Note on Figure 3B that the difference between the effects of SAR against TMV and CMV infection did not show significant difference. This feature indirectly excludes the activation of a virus species-specific mechanism during SAR induction. Furthermore, effectiveness of TMV-induced SAR against bacteria (Figure 3C) was comparable to bacterial induced SAR response in tobacco and *Arabidopsis* [21,42].

Viral replication is generally associated with the presence of double stranded RNAs (dsRNAs) [43,44]. Synthetic analogues and viral dsRNA itself act as MAMPs and can induce SERK-1 dependent specific antiviral resistance signalling in *Arabidopsis* but this mechanism is independent of RNA silencing [45]. The significance of this fact in SAR induction is unknown. Furthermore, PTI responses were not impaired in plants lacking the two main antiviral dicer-like (DCL) proteins, DCL2 and DCL3 [46] and the short interfering RNA (siRNA)-producing DCL3 [47]. These results also suggest no role of silencing mechanisms during PTI. Moreover, bacterial RNA can also induce PTI-mediated plant defence [48].

A further example of the non-specific nature of SAR response is the accumulation of PR proteins and especially PR1. PR1 is upregulated in SA-dependent manner after microbial (fungal, bacterial or viral) infections and used as marker gene for SA-mediated disease resistance and SAR. Several groups have demonstrated that overexpression of *PR1* in transgenic plants result in increased resistance to oomycetes, fungi, bacteria but not to viruses (for review see [49]). Recently Gamir et al. [50] presented a link between anti-oomycete activity and sterol (especially fungal ergosterol) binding capacity of PR1a from tobacco and P14c from tomato via CAP (sterol binding) domain of PR1 proteins. Moreover, the addition of fungicide that blocks sterol biosynthesis in sterol prototrophic fungi, such as *Aspergillus niger* and *Botrytis cinerea*, made these fungi susceptible to P14c. This model suggests that PR1 proteins have antimicrobial function by sequestering sterols from the membranes of microbes and that they are more effective against sterol auxotrophic organisms (for example oomycetes) which are dependent on sterol supply from the environment. Besides this feature, PR1 proteins harbour an 11-amino acid peptide (CAP-derived CAPE1) at the C terminus with conserved consensus motif PxGNxxxxxPY. CAPE1 peptide can induce an antibacterial effect [51]. Interestingly, CAPE1 did not stimulate the expression of WRKY transcription factor 53 (WRKY53) which is highly upregulated by bacterial MAMPs. Further studies are required to test the putative role of this PR1-derived peptide in the antibacterial property of SAR induction. Furthermore, the lack of antiviral effects of PR1 could be replaced by direct antiviral action of SA on virus replication [52].

Taken together, all of these examples argue for the specificity of pathogen signal recognition/perception and aspecificity of the induction of SAR response in plants.

This review will focus on signal transduction mechanisms between pathogen perception and SAR response, especially on the environmental factors that can influence, qualitatively or quantitatively, the signalling processes.

## 2. Multiple Signalling by Chemically Diverse Compounds during SAR Induction

### 2.1. DIR1 (DEFECTIVE IN INDUCED RESISTANCE1)

One of the SAR signal transduction related compounds, DIR1 protein, is involved in the signalling function of other signal molecules (AzA, DA, G3P, MeSA) as a lipid transfer protein (LTP). Therefore, DIR1 will be covered here, before the other chapters. The *dir1* locus was identified in classical mutational analysis as SAR-deficient phenotype [14].

Later on, besides DIR1, DIR1 homolog DIR1-like proteins were also identified in *Arabidopsis* [53,54]. Additional LTPs, like LTP1 and LTP2 and even AZELAIC ACID INDUCED1 (AZI1) and its homologs have a role in SAR signal development [54,55]. Moreover, DIR1 is conserved in other SAR-competent species like cucumber [56] and tobacco (*Nicotiana tabacum*) [57]. The lipid/acyl-CoA binding protein, ACBP6 is also required for SAR induction [58] and present in phloem exudates of SAR induced *Arabidopsis* plants [59].

The small 7 kDa DIR1 protein is present in the petiolar exudate of infected leaves as a high molecular mass oligomeric form or in a complex with other proteins [16]. On the other hand, SAR-inducing capacity of petiolar exudate of infected leaves was proteinase sensitive [16,20]. The overexpression of two *Arabidopsis* proteins, localized in the plasmodesmata, results in the inhibition of the movement of DIR1 protein and SAR induction suggesting a symplastic transport [15]. The effect of external AzA application was DIR1-dependent [17]. Similar results were reported for DA [16] and G3P dependent factor [20]. DIR1 may also interact with MeSA signalling in distant leaves of tobacco [57].

### 2.2. Salicylic Acid (SA) and Methyl Salicylate (MeSA)

Salicylic acid (SA) and its volatile derivative, methyl salicylate (MeSA) were the first two candidates for signalling of SAR (Figure 1 and Figure 2A). In the latter case MeSA was suggested either as a volatile, intra-plant airborne signal or a long-distance inside signal transported via the phloem system.

The first reports in different plants (tobacco and cucumber) strongly suggested that SA could be required either for SAR signalling or development of SAR in the distant leaves [60,61,62]. These proofs were as follows: (i) SA is accumulated not only in the infected leaves but in distant pathogen-free leaves; (ii) SA is accumulated both in infected leaves and their phloem (petiolar) exudates; (iii) in transgenic plants expressing constitutively a salicylate hydroxylase (*NahG*) gene the SAR response was absent; (iv) externally applied SA was translocated and resulted in SAR-like response [63]. In addition to the effect of *NahG* gene (where there is no SA accumulation), some other biochemical or genetic approaches led to the same conclusion. For example, the inhibition of SA biosynthesis also affected the SAR response. Two *Arabidopsis* mutants, *sard1* (systemic acquired resistance deficient1) and *cbp50g* (calmodulin binding protein 50g) also have SAR-deficient phenotypes. The corresponding proteins are involved in the regulation of SA-biosynthesis via *ICS1* (*ISOCHORISMATE SYNTHASE1*). These mutant proteins were unable to bind to the promoter region of *ICS1* gene (its protein product is a key enzyme of SA biosynthesis localized in the chloroplasts) after infection [64,65]. Plants with *sid1* and *sid2* (salicylic acid induction deficient) mutant loci (the latter is responsible for coding of ICS1 in *Arabidopsis*) were also unable for SA-biosynthesis and induction of SAR [66,67,68]. Somewhat similar to the effect of *NahG*, *OsBSMT1* (*BENSOIC ACID/SALICYLIC ACID CARBOXYL-METHYLTRANSFERASE1* in rice) gene is also involved in the regulation of free SA accumulation. Transgenic overexpression of this gene resulted in the inhibition of local SA-accumulation (via formation of MeSA from SA) and caused susceptibility to bacterial and fungal pathogens [69]. In tobacco (*N. tabacum*) and cucumber, however, SA accumulation was blocked by biochemical inhibition of PAL (PHENYLAMMONIA LYASE) enzyme and inhibition of *PAL* gene expression [70,71] indicating a putatively different route for SA-biosynthesis. In *N. benthamiana*, however, *ICS1* gene was responsible for SA-accumulation both after biotic and abiotic stress [72].

All of these results, whatever kind of the biochemical pathway for SA biosynthesis is, undoubtedly proved the important role of SA in local resistance and in the induction of SAR, but shad no or little light on the putative signalling function of SA. The first question mark in this context was related to the report on the activation of de novo SA biosynthesis in systemic, pathogen-free tissues [73]. Thus, the only source of systemic SA accumulation could not be the long-distance SA (or MeSA) transport from infected leaves. However, this result does not yet exclude the role of SA in signal transduction of SAR. Later reciprocal grafting studies in tobacco between transgenic *NahG* (deficient in SA-accumulation) or *PAL*-silenced plants (partially deficient in SA-synthesis) and WT plants, indicated a more direct conclusion: (i) the rise in free SA content in primary, pathogen-inoculated leaves is not critical to the induction of SAR in distant leaves; (ii) the long-distance signal is not identical to SA, in spite of the accumulation of SA in petiolar exudate of primary inoculated leaves; (iii) the accumulation/presence of SA in distant, pathogen-free leaves is required for the induction of SAR; (iv) different factors are responsible, at least in part, for local and systemic resistance responses [71,74]. Thus, it is important to note that SA plays differential role in local and systemic resistance responses.

As indicated above, systemic SA accumulation is required for the induction of SAR. In *Arabidopsis*, upregulation of *ICS1* gene expression and de novo SA-biosynthesis in distant leaves is also required for SAR induction [75]. Further experiments with *Arabidopsis sid1* and *sid2* and the respective double mutants, however, suggest a modest SAR induction, indicating an SA-independent route, in addition to a dominant SA-dependent SAR activation pathway [23]. Further support for de novo SA-biosynthesis in the distant, pathogen-free leaves comes from the characterisation of two SAR-deficient mutants, *fld* and *fmo1* [76,77]. Surprisingly, SA content increased in primary pathogen inoculated leaves and the SAR signal was systemically transported in *fld* mutants. On the contrary, distant leaves of *fld* mutants did not accumulate SA. Since FLD affects histone modifications, it is likely that FLD-dependent changes in histone modifications at genes involved in SA accumulation/metabolism are associated with SA-dependent SAR induction mechanisms [76]. Systemic accumulation of SA and SAR induction were also attenuated in the *fmo1* mutant *Arabidopsis* plants and *FMO1* was suggested to be a part of SA amplification loop including Pip [21,77].

Besides free SA, several other compounds are involved in SA metabolism. For example glycosylated SA compounds, like SA-glycosyd (SAG) are also accumulated after infection [75,78]. The methylation of free carboxyl group of SA (2-hydroxy benzoic acid) results in the formation of a volatile compound, methyl-salicylate (MeSA) [79].

The role of MeSA, as a volatile and airborne signal in SAR induction was first suggested by Shulaev et al. [11]. In a closed artificial system infected plants (used as MeSA donor plants) could produce enough amounts of MeSA to induce about 30% decrease in TMV lesion size of acceptor plants.

Later, reciprocal grafting experiments between WT and *SAMT1* (*SALICYLIC ACID METHYLTRANSFERASE1*) silenced tobaccos, however, suggested that MeSA can serve as a signal for SAR induction inside the plants via transport [12]: (i) functional *SAMT1* gene was necessary for SAR induction in primary inoculated leaves; (ii) MeSA accumulated in phloem exudate of infected leaves and thus MeSA could be the long-distance signal for SAR; (iii) competitive, pharmacological inhibitor of MeSA demethylase, 2,2,2,2-tetrafluoro-acetofenon could attenuate SAR development in the distant, pathogen free leaves and finally (iv) further grafting experiments between WT and *SABP2* (*SALICYLIC ACID BINDING PROTEIN2*) silenced plants also suggested the importance of its demethylase function in distant leaves in SA production and SAR induction [12,80]. This scenario was later expanded to other plants, *Arabidopsis* [81,82] and potato [13]. This model suggests that SA is converted to MeSA in primary inoculated leaves and then MeSA is translocated to distant leaves, inside the plants and finally converted to SA and SA accumulation in systemic leaves results in SAR induction [83].

However, there is a controversial step during either the airborne or inside signal function of MeSA [28]. If SA accumulation is inhibited in *NahG* rootstock plants but grafting with WT plants results in SAR induction [74], this conclusion may exclude the possibility that MeSA could be produced from SA in the primary inoculated leaves and subsequently serves as a signal for SAR induction (considering that only one pathway is responsible for MeSA production and this pathway is identical to the production of MeSA from SA). This controversy, however, will be addressed in chapter 3, in connection with the effect of the length of light exposition after primary inoculation on SAR signalling [84].

In fact, Attaran et al. [75] found that *bsmt1* mutants in *Arabidopsis* could induce SAR response, similar to WT plants, in spite of the fact that these plants could not accumulate and evaporate MeSA in/from the primary inoculated leaves. These results suggested that MeSA has role neither in SAR signalling nor in systemic SA accumulation. Four more points were listed against the role of MeSA signalling in SAR induction and generally against volatile signals including MeSA: (i) blockage of phloem transport attenuates SAR induction [85,86]; (ii) the volatile concentration of MeSA (10–1000 µg L^−1^) required for the induction of resistance in closed systems is two magnitude of orders higher than the concentration that could be present under normal conditions in phytotron chambers; (iii) MeSA was present in very low amounts in petiolar exudates of infected leaves and contrary to SA, MeSA did not accumulate in pathogen-free systemic leaves and finally (iv) ICS gene expression and de novo SA biosynthesis are required for systemic SA accumulation both in WT and *bsmt1* mutant plants suggesting no role of airborne MeSA and/or MeSA transported via the phloem system [74]. Recently, the role of other volatile compounds, monoterpenes (α-pinene and β-pinene) was reported in SAR via induction of reactive oxygen species (ROS) and *AZI1* gene [87].

This controversy of results may indicate that MeSA signalling is influenced by unknown environmental factors and in this case other signal molecules could be responsible for SAR signal transduction.

### 2.3. Lipid-Derived Signalling

#### 2.3.1. Glycerol-3-Phosphate Dependent Factor (G3P-Dependent Factor)

G3P is an important intermediate of lipid biosynthesis and is present both in the cytosol and chloroplast (Figure 2C). Mutational analysis revealed that functional *SFD1* (*SUPPRESSOR OF FATTY ACID DESATURASE DEFICIENCY1* in Nössen genotype) locus was necessary for SAR induction [19]. The same locus is also known as *GLY1* from another *Arabidopsis* genotype [88]. *SFD1* gene codes for a dihydroxy acetone phosphate (DHAP) reductase enzyme, which is responsible for the synthesis of G3P from DHAP [19,89]. In *sfd1* mutant *Arabidopsis* plants, the SA accumulation and *PR1* expression were reduced in distant, pathogen-free leaves but the local response to pathogen infection was not modified. The *sfd1* mutation influences fatty acid composition of galactolipids in chloroplasts, especially the ratio of C16:3 in monogalactosyldiacylglycerol (MGDG) and digalactosyldiacylglycerol (DGDG) is decreased [89]. However, the mode of action of SFD1 is unclear as this protein has no desaturase activity.

The chloroplastic localisation (encoded by an N-terminal signal sequence) and the enzymatic DHAP-reductase activity of SFD1 were necessary for SAR induction [89]. Petiolar exudate from WT plants induced resistance in *sfd1* mutant plants but the opposite experiment gave negative results suggesting a role for G3P and/or a G3P dependent factor in signalling of SAR [90].

To clarify the real function of G3P in SAR signal transduction, other lipid biosynthesis related mutants were also tested. Fatty acid desaturase (*fad7* and *sfd2*) and *mgd1* (monogalactosyl synthase 1, responsible for galactose incorporation into diacylglycerol) mutants had also SAR-deficient phenotype. Therefore, it was concluded that rather the intactness of chloroplastic glycerolipid biosynthesis is required for SAR but not the production of C3 carbon skeleton [90].

Interestingly, another gene mutation, *gli1*/*nho1* (glycerol insensitive 1/nonhost 1) involved in G3P biosynthesis in the cytosol, also has a SAR-deficient phenotype. The enzymatic glycerol kinase activity of GLI1/NHO1 protein produces G3P from glycerol [20]. Therefore, the effects of these two mutations (*gli1*/*nho1* localized in the cytosol and *gly1* in the chloroplast) on fatty acid composition of lipids were compared. Surprisingly, *gli1* mutation has no effect on lipid composition, contrary to chloroplastic *gly1* (see earlier results with *sfd1*, which is allelic to *gly1*) [20]. Moreover, the SAR was also inducible in the *act1* (G3P acyltransferase1) mutant, in which the incorporation of C18:1 fatty acid into glycerolipids and other aspects of lipid biosynthesis are inhibited. However, there are three more genes in *Arabidopsis* with DHAR reductase activity. Independently of their subcellular localization, these genes had no effect on lipid composition, but one cytoplasmic and one chloroplastic isoform affected SAR development. Therefore, contrary to former results [90], it was concluded that intactness of glycerolipid biosynthesis is dispensable for SAR induction and the regulation of G3P level is a crucial factor.

Taken together, *sfd1/gly1* and *gli1* mutations in *Arabidopsis* indicated that the effects of these two mutations on glycerolipid biosynthesis are different, but the SAR-deficient phenotypes in both cases could be related to their contribution to G3P synthesis and production of a G3P dependent factor. Supporting this evidence, after infiltration of ^14^C-G3P into primary inoculated leaves, this compound was not transported to distant leaves, but was present as an unidentified labelled compound in systemic leaves suggesting a signalling role of a G3P-dependent factor [20]. Importantly, localized application of G3P induces transcriptional changes in distant tissue indicating a role for G3P in systemic transcriptional reprogramming [20].

As indicated above, galactolipids may play an important role in lipid-derived signalling of SAR. The role of two galactolipid mutants *mgd1* and *dgd1* (digalactosyl synthase 1), responsible for MGDG and DGDG synthesis, respectively, in SAR induction was also studied in detail by Gao et al. [90]. Both mutants were compromised in SAR induction. Although the two mutants had differential and multiple impacts on SAR signalling capacity (*mgd1* plants are impaired in pathogen-induced ROS, AzA and G3P accumulation, but *dgd1* mutants in nitric oxide (NO) and SA synthesis), surprisingly these mutants were able to produce biologically active petiole exudate to induce SAR in WT plants. To explain these results one possibility is that these mutants can produce other active signalling compounds but are unable to respond to them. Thus, MGDG and DGDG lipids are rather involved in signal perception than in signal generation. Transgenic expression of a bacterial glucosyltransferase is unable to restore SAR in *dgd1* mutant plants even though it can rescue their morphological and fatty acid phenotypes [91].

#### 2.3.2. Azelaic Acid (AzA)

Azelaic acid is a nine-carbon dicarboxylic acid (1,9-nonanedionic acid) (Figure 2E). In human medical and cosmetic practice, AzA is used for treatment of different types of hyperpigmentation, acne and other skin disorders [92]. In plants, AzA is an end-product of lipid peroxidation (LP) under biotic (especially during hypersensitive reaction (HR) caused by incompatible plant-pathogen interactions) and abiotic stress conditions and is produced via different enzymatic and non-enzymatic mechanisms [93].

Considering AzA as a signalling molecule in SAR induction in *Arabidopsis* Jung et al. [17] found that although AzA accumulated at elevated levels locally and in phloem exudates (6–7 times) during bacterial induced SAR, external application of AzA per se did not promote SA accumulation and had a minimal effect on gene expression but could induce local and systemic resistance response. AzA can act as a priming molecule and produces elevated systemic induction of SA accumulation upon bacterial inoculation (*P. syringae* pv. *maculicola* strain *Pma*DG3) of distant leaves with enhanced resistance against the pathogen. Moreover, AzA-induced SAR was dependent on *FMO1* and *ALD1* [17] both of which are involved in the amplification loop of systemic SA and Pip accumulation [21] (see later). Mutation of the *AZI1* gene, which is inducible by azelaic acid, results in the specific loss of systemic immunity triggered by pathogen or AzA [17]. Yu et al. [18] studied the role of fatty acids in SAR induction. Local treatment of *Arabidopsis* leaves with C18 unsaturated fatty acids and its release after bacterial inoculation can serve as precursors of AzA production and trigger SAR induction. AzA in turn can increase G3P accumulation and expression of lipid transfer proteins, DIR1 and AZI1. Wittek et al. [94] also reported that EDS1-dependent SAR is mediated in an AzA- and (its precursor) 9-oxo nonanoic acid-dependent manner in *Arabidopsis*. Metabolomic studies in tobacco cell cultures indicated that AzA treatment can induce the accumulation of the early products of the phenylpropanoid pathway [95]. The putative consequence of this finding on resistance was not tested. On the contrary, Návarová et al. [21] found no increase in accumulation of AzA in petiolar exudate of bacteria (*P*. *syringae* pv. *maculicola, Pma*)-infiltrated *Arabidopsis* leaves. Later studies suggested that AzA can locally induce SAR signal(s) emission in primary infected leaves via AZI1 and its paralog, EARLI1 (EARLY ARABIDOPSIS ALUMINIUM INDUCED1) accumulation at membrane-membrane contact sites (MCS) required for intracellular transport of apolar lipidic signals including AzA [55]. It is important to emphasize that inducible expression of *AZI1* or *EARLI1* only in local tissue of *az1* mutant plants is sufficient to restore SAR [55]. Moreover, an interesting finding is that AZI1:GFP protein was mainly detected in epidermal cells [55]. Importantly, the plastids of these cells are specialized organelles in which fatty acids and cuticle components for the epidermal cell surface are synthesized. The fact an intact cuticle is needed for SAR [58] supports the idea that epidermal cells may be important for long-distance defence signalling.

Therefore, more recently, our own studies have re-examined the role of AzA in SAR induction, in another plant-pathogen system, tobacco mosaic virus (TMV)-tobacco (*N. tabacum* cv. Xanthi nc) [31]. Former results indicated that signal transduction from inducing leaves into distant ones is fully completed within 4 days after primary TMV inoculation [28,30]. Therefore, phloem sap was collected in this time window (2 or 3 days after inoculation) for 24 h from TMV-infected and control leaves. Interestingly, HPLC-MS assays detected (besides C9 AzA) low amounts of two other dicarboxylic acids, suberic acid (1,8-octadienoic acid), and sebacic acid (1,10-decadienoic acid) in petroleum ether extracted petiolar exudates of both TMV-infected and control leaves 2–3 days after TMV inoculation. According to Jung et al. [17] these two other dicarboxylic acids had no biological activity in SAR induction. AzA content was doubled and significantly higher in exudates of TMV-infected leaves as compared to that in control exudates [31]. In our experiments, we focused on the effects of external AzA application on symptom expression. Local and systemic effects of AzA pretreatments on the distribution of TMV lesion size were measured by a semi-automated, computer-assisted method and significance of the results were analysed by a multiple comparison test (R package) [96]. The local application of AzA (0.2–1.0 mM) showed no or limited influence (either increase or decrease) on lesion size of TMV-inoculated leaves. In addition, AzA pretreatment did not modify the multiplication of TMV detected by semiquantitative RT-PCR of coat protein gene. No significant systemic effect of AzA on lesion size of TMV was detectable in distant leaves. Moreover, AzA treatment had no considerable local and systemic effect on symptom expression and multiplication of incompatible (*P*. *syringae* pv. *tomato* DC3000, *Pst*) and compatible (*P. syringae* pv. *tabaci*) bacteria [31].

In accordance with our results, Zoeller et al. [93] found that in spite of the bacterially inducible AzA accumulation in infected leaves, external AzA treatment does not inhibit the growth of *Pst* (strain DC3000) in *Arabidopsis* leaves. Vicente et al. [97] also found that AzA pretreatment caused a barely detectable inhibition of symptoms and growth of *Pst* DC3000 bacteria in both treated and distant *Arabidopsis* leaves.

Some other aspects of the mode of action of AzA in SAR induction have also been discussed previously. Unlike the priming effect of AzA for pathogen-responsive SA biosynthesis in distant *Arabidopsis* leaves [17], other studies [18] did not find any priming of SA accumulation in systemic leaves. The biosynthesis of AzA is a complicated question. Although the 9-lipoxygenase (9-LOX) pathway is involved in many plant defence responses against bacterial, fungal and viral infections [97] and produces mostly nine-carbon products, Zoeller et al. [93] reported that bacterium-induced AzA is synthesized non-enzymatically from chloroplastic galactolipids. It is more likely that AzA was synthesized via ROS-mediated pathway from chloroplastic galactolipids (MGDG and DGDG) [93,98]. In addition, in *lox1 lox5* double mutant *Arabidopsis*, pathogen-induced AzA production was not compromised suggesting that *9-LOX* genes are not required for AzA biosynthesis. More importantly, *lox1* (*9-LOX*) mutant *Arabidopsis* plants were not able to develop fully active SAR indicating a putative role of 9-LOX-mediated lipid signal generation in primary inoculated leaves and/or signal manifestation in distant leaves [97].

Taken together, our and some other [21,93,97] results suggest the lack of or limited accumulation of AzA after infection and external AzA treatment does not induce considerable local or systemic effects on viral and bacterial infections. Therefore, its formerly reported role in signal transduction and/or signal generation during induction of SAR in *Arabidopsis* could not be confirmed under our experimental conditions in tobacco [31]. However, it is possible that the role of lipid-derived signalling could be relatively less pivotal under certain conditions as SAR induction is considered independent of tissue necrotisation [39] that is associated with LP.

### 2.4. Dehydroabietinal (DA)

One of the most potent SAR inducers is dehydroabietinal (Figure 2D). This tricyclic diterpenoid compound can induce SAR in picomolar ranges when applied to leaves in *Arabidopsis*, tomato and tobacco [16,99]. In conifers, these abiatene diterpenoids are synthetised from geranylgeranyl diphosphate by cyclization. *Arabidopsis* contains a homolog of a cytochrome monooxigenase P450 class enzyme of *Picea sitchensis* that is capable of synthesizing DA and related compounds from dehydroabiatadiene [100].

SAR induction by DA has common and specific features as compared to other signalling compounds. Like pathogen-induced SAR and the mode of action of Pip (see later), DA requires functional *FMO1* and *ICS1* genes for systemic SA accumulation and SAR induction in distant leaves of *Arabidopsis* [16]. Despite that pure labelled ^2^H-DA is rapidly translocated from treated leaves to the foliage and induced SAR, DA levels did not increase in leaves and petiolar exudate after infection of leaves with a SAR-inducing pathogen. However, due to the infection DA is enriched from a biologically inactive low molecular weight fraction into a trypsin-sensitive high molecular weight signalling form (DA*, 100 kDa) that is capable of SAR induction [99]. It is, however, unclear whether the pure application of DA to leaves also leads to this activation and if so, which factor is responsible for this activation process under pathogen-free conditions. Anyway, DIR1 which could be systemically translocated was associated with the high molecular weight DA* fraction [16,99]. Indeed, DIR1 was required for the full activation of SAR by DA, confirming an important function of DIR1 in DA-induced SAR.

The application of DA also promotes flowering in *Arabidopsis*. Shortly, during vegetative growth, FLC (FLOWERING LOCUS C), a flowering repressor protein suppresses the expression of flowering signal *FLT* (*FLOWERING LOCUS T*) which is considered to be the phloem-mobile florigen and released from leaves and transported to the shoot meristem to induce transition from vegetative to generative state. DA and bacterial inoculation both promote expression of *FLD* (*FLOWERING LOCUS D*) which involved in histone modifications. FLD promotes flowering by suppressing the expression of flowering repressor *FLC* gene [101]. FLD function is also required for systemic SA accumulation and priming of *PR1*, *WRKY6* and *WRKY29* expression in distant leaves [76,102].

### 2.5. Pipecolic Acid (Pip) and N-hydroxypipecolic Acid (NHS)

l-pipecolic acid is an enigmatic heterocyclic non-protein amino acid (Figure 2B) and a catabolite of l-lysine (Lys). In humans it serves as a diagnostic marker of pyridoxine-dependent epilepsy [103] and accumulates in patients with hyperpipecolic acidemia (hyperpipecolatemia), a rare, recessive metabolic disorder related to peroxisomal malfunction [104]. This derivative was also present among others in non-protein amino acids of an extraterrestrial meteorite [105].

In plants, especially in angiosperms, the level of Pip is elevated in a response to different stresses including pathogen infection [106]. Although former genetic studies with an *ald1* mutant indicated the key role of an aminotransferase, ALD1 in local and systemic defence responses [24,107], the function of Pip was discovered only later on [21]. In fact, detailed studies indicated that (i) *ALD1* gene product shows in vitro substrate preference to lysine, a putative precursor of Pip biosynthesis in plants and animals [97,107]; (ii) the biosynthesis of Pip in *Arabidopsis* is dependent on functional *ALD1* locus [21] and (iii) ALD1 enzyme acts as a first step during Lys catabolism and directly transfers the α-amino group of l-Lys to an oxoacid, preferentially pyruvate to form ε-amino-α-ketocaproic acid (KAC) and alanine [22,108]. Next steps from KAC (cyclization, isomerization) via 1,2-dehydropipecolic acid and its in planta detectable, enaminic form, 2,3-dehydropipecolic acid are leading to the formation of Pip [22]. Furthermore, *ALD1* transcript accumulates in the pathogen-inoculated and distant pathogen-free leaves [107]. The local and systemic immune defects of *ald1* mutant *Arabidopsis* after bacterial inoculation could be rescued by external application of Pip. From the point of view of signal transduction during SAR response, it is important to note that Návarová et al. [21] found strong Pip accumulation in petiolar exudate of SAR-inducing *P. syringae* infected leaves. However, whether Pip has a direct role in long-distance SAR signalling remains to be elucidated in the future. Recent results show that transcription factors TGA1 and TGA4 (TGAGG-BINDING FACTOR) also regulate Pip and SA synthesis by modulating the expression of *SARD1* and *CBP60g* genes [109].

**Figure 4:**
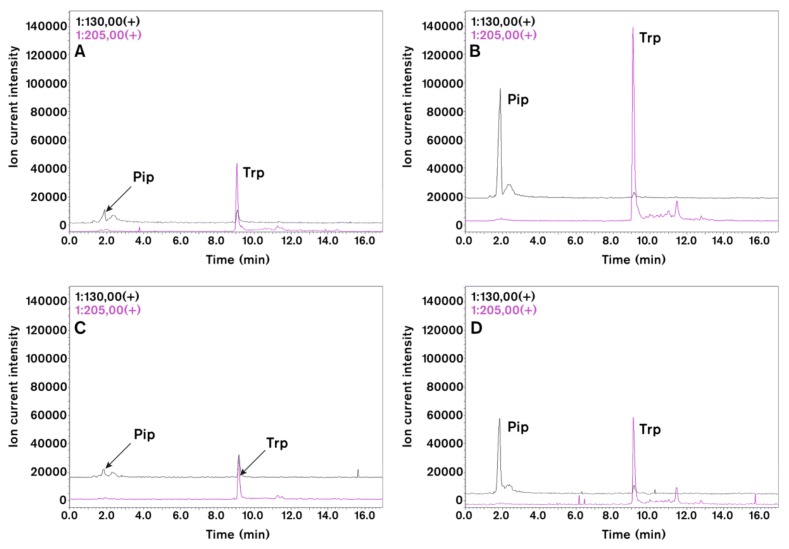
Effect of TMV (**A**,**B**) and CMV (**C**,**D**) inoculations on selected amino acids of tobacco leaves (4 days after viral inoculation). HPLC-MS chromatograms of pipecolic acid (Pip, *m*/*z* = 130) and tryptophan (Trp, *m*/*z* = 205) in virus infected leaves (**B**,**D**) as compared to control plants (**A**,**C**). Leaf samples (about 200 mg) were extracted with acetonitrile:water (6:4 v/v in 1.5 mL). This condition supports amino acid extraction but limits protein solubilization. HPLC-ESI-MS analyses were carried out with a Shimadzu LCMS-2020 (Shimadzu Co., Kyoto, Japan) analytical system. Chromatographic separations were performed on a SunShell C18 packed column (2.6 µm, 3.0 mm × 100 mm) by using gradient elution (solvent A, 0.1% formic acid solution; solvent B, 0.1% formic acid in acetonitrile) under non-derivatised conditions. Ions for MS detection were obtained by positive mode of electrospray ionization (ESI). Identity of peaks were also checked by co-chromatography of samples with authentic standards (Sigma-Aldrich Co. St. Louis, MI, USA).

**Figure 5:**
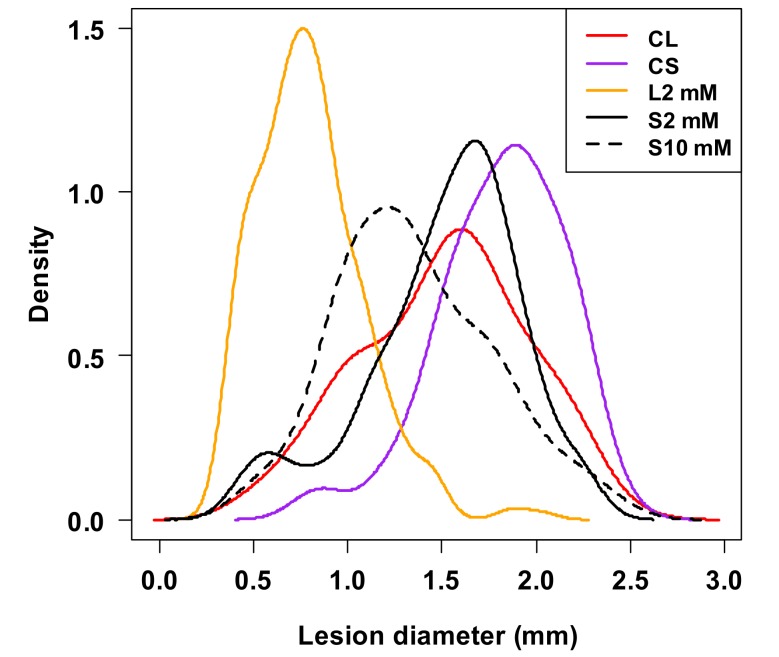
Effect of local (CL and L2) and systemic (CS, S2 and S10) pipecolic acid (d-l-Pip) treatments on TMV lesion size distribution by using Kernel density estimation [31,32] (2 days after Pip infiltration). L2, S2 and S10: local (L, leaf 6) and systemic (S, leaf 7) effects of 2 and 10 mM d-l-Pip treatments as compared to corresponding local control (CL) and systemic control (CS) leaves (triplicate per treatments). In a multiple comparison test [31], both concentration of Pip treatment resulted in significant decrease of TMV lesion size. The highest difference was caused by local treatment (L2). The effect of systemic treatment with 10 mM Pip was more pronounced (and significantly different) than with 2 mM Pip

To prove further the conserved role of Pip in plant immune responses and to analyse further virus-induced SAR signalling in tobacco we measured by HPLC-MS the level of different amino acids in virus infected tissue (Figure 4) [40]. Both in TMV and CMV infections the level of Pip and tryptophan (Trp) accumulated to high amounts. Chromatograms on Figure 4 indicate about tenfold increase in Pip level after TMV (Figure 4A,B) and CMV (Figure 4C,D) infections. We also analysed the local and systemic effects of external Pip infiltration (2–10 mM d-l-Pip) into tobacco leaves (Figure 5) [40]. Interestingly, Pip had not only local but systemic effect. Comparison of TMV lesion size distribution in locally treated leaf 6 and systemic leaf 7 to corresponding controls indicated significant reduction in lesion size. This effect, however, was less pronounced (especially after local treatment with 2 mM Pip) in systemic leaves, but even this effect was comparable to the effect of SAR induction by TMV infection (see Figure 3A). Návarová et al. [21] found that exogenous application of Pip via root system is sufficient to induce SAR-like response and primed state in wild type *Arabidopsis* against bacteria. Consequently, Pip can be an important player in SAR induction in different plants against different pathogens.

In two very recent publications, added in proof, a new SAR signalling compound, FMO1-dependent N-oxygenation product of Pip, N-hydroxypipecolic acid (NHS) was described (Figure 1) [110,111].

## 3. Role of Light in SAR Induction: Light Intensity, Timing of Exposition and Spectral Distribution

Former results have already indicated the important role of light in plant defence responses and especially in SAR induction. New insights into this question show that not only effectiveness of SAR induction is regulated by light dependent factors, but the quality of signalling compounds. There are several light dependent factors that can influence SAR signalling events. Besides light intensity and timing of illumination after primary inoculation, a third factor, the spectral distribution of light will be also taken into consideration.

Under high light intensity (over 500 μE m^−2^ s^−1^) SAR can develop without SA accumulation and PR1 expression in distant leaves [112]. This pathway, however, depends on the expression of *FMO1* required also for many other signalling routes during SAR induction (see earlier). The systemic expression of *FMO1* gene depends on phytochromes [76,113]. Earlier results suggested that hypersensitive necrotisation and local resistance response in *Arabidopsis*–*Pst* AvrRpt2 interaction depended on phytochrome A and B [114]. Griebel and Zeier [113] reported that rather the systemic response depends on phytochromes in *Arabidopsis*–*Psm* AvrRpm1 interaction. The systemic *FMO1* and *PR1* expression and SA level were not inducible as well as SAR could not develop in the double mutant *phyA*-*phyB* plants. It is possible that the phytochrome mediated effect is related to the effect of spectral distribution of light on SAR [32].

The interconnection between chloroplastic photoreceptor function and pathogen signalling is suggested by the similarity of photooxidative stress response (ROS generation, PR1 expression and programmed cell death) that can induce resistance against compatible bacteria in local and systemic tissues [115]. In accordance with this finding Fodor et al. [116] found that SAR induction by TMV infection in tobacco was associated with elevated antioxidant capacity of distant resistant leaves.

Local resistance responses (hypersensitive reaction, PR1 expression and SA accumulation) could be inhibited if plants were kept in darkness after infection for a longer period [110,117]. The development of SAR was also inhibited in *Arabidopsis* after exposition to darkness all over the experiment [112]. If plants (*Arabidopsis* and tobacco) were kept in darkness only for overnight period after primary inoculation, the SAR response was present but became weaker as compared to the SAR response of the plants that were exposed to light (at least 3.5 h) after inoculation [84]. The longer light exposition after primary inoculation correlated with the level of SA accumulation, PR1 expression and the strength of SAR response in *Arabidopsis*–*Psm* AvrRpm1 plant-pathogen system [113].

The discrepancy of the results in signal transduction of MeSA during SAR induction was also explained by the effect of light exposition after primary inoculation [84]. Later studies indicated that several hours (at least 3.5 h or more) of light exposition after primary inoculation with bacteria or TMV restore the SAR-deficient phenotype of *bsmt1* (responsible for MeSA synthesis) mutants [84]. Moreover, two other signal transduction mutants of SAR, *dir1-1* and *sfd1/gly1* mutants were also complemented under this light condition. In other words, the timing of the dark period relative to the primary inoculation severely influences the importance of a certain signal transduction component in SAR induction [57,84].

However, in our experiments with AzA, plants were exposed to light for at least 10 h after treatments and were illuminated with a relatively high daily photon flux. Therefore, we performed experiments with tobacco plants kept in darkness subsequent to AzA treatment to test whether this condition can activate AzA-mediated local and/or systemic response in tobacco. The local AzA treatment did not show significant difference on TMV lesion size of local or systemic leaves after incubation in darkness as compared to control plants. Experiments with the multiplication of a compatible bacterium, *P. syringae* pv. *tabaci*, in local and systemic leaves after AzA treatment in darkness also showed no significant decrease. These data clearly suggest that AzA-mediated signalling does not rely on factors activated in darkness, at least in tobacco plants [31].

The third light-dependent factor that can cause differences in resistance to TMV infection is the spectral distribution of light [32]. Our results clearly indicated that spectral distribution of light sources influences (i) plant growth and development; (ii) local resistance response to TMV infection and (iii) SAR inducing capacity of tobacco plants. Certain light sources with unbalanced light spectrum had negative impact on plant growth and development, local resistance response and SAR induction capacity of tobacco plants. Halogen lamp (HL) and fluorescent tube (FT) light sources showed very different spectral distribution, relative abundance or shortage in red/far red light, respectively. The more similar was the spectrum of the artificial light source to sunshine (greenhouse conditions), the stronger was the inducible SAR response. From a practical point of view, under artificial conditions, metal halide lamp or a mixture of HL and FT light sources can be suggested as optimal test conditions. Consequently, the optimization of the effect of artificial light sources is an important factor in experimental design studying signal transduction and biochemistry of SAR [32].

## 4. Concluding Remarks

At least three separate signalling pathways are present in plants to induce SAR. To date, these pathways could be discriminated by different light conditions indicating the importance of this environmental factor and putatively the functional role of chloroplasts in signal generation. The SAR signalling routes and their main characteristics are as follows: (i) Under high light intensity conditions, SAR is induced without systemic SA accumulation but requires functional *FMO1* gene. The signal is unknown but could be related to ROS-mediated processes; (ii) If after the primary inoculation plants are exposed to a longer (overnight) dark period, SAR induction and signalling may depend on *BSMT1* (MeSA), *DIR1* and *SFD1/GLY1* genes and systemic SA accumulation; (iii) If the SAR induction is dependent on a light period after primary inoculation, it requires functional *ICS1*, *FMO1* and *ALD1* genes, Pip and SA accumulation in the systemic leaves. Pip and its FMO1-dependent N-oxygenation product, *N*-hydroxypipecolic acid can play a critical role in the induction of SAR either after bacterial or viral infection in different host plants. Finally, (iv) DA-mediated signalling is *DIR1*-, *ICS1-*, *FMO1-FLD*- and SA-dependent in systemic leaves, but its relation to light was not yet determined. Optimal SAR induction also requires balanced spectral distribution of light, probably due to phytochrome regulation.

Parallel operation and control of different signals probably can contribute to the plasticity of the SAR response. However, further detailed analysis of the interaction of these overlapping factors is required for the practical application of this resistance mechanism for protecting field crops.
